# Duplex Droplet Digital PCR Assay for Quantification of Hepatitis E Virus in Food

**DOI:** 10.3390/v16030413

**Published:** 2024-03-07

**Authors:** Gianfranco La Bella, Maria Grazia Basanisi, Gaia Nobili, Anna Mattea D’Antuono, Elisabetta Suffredini, Giovanna La Salandra

**Affiliations:** 1 Istituto Zooprofilattico Sperimentale della Puglia e della Basilicata, Via Manfredonia 20, 71121 Foggia, Italygaia.nobili@izspb.it (G.N.); teadantuono@libero.it (A.M.D.); giovanna.lasalandra@izspb.it (G.L.S.); 2 Department of Food Safety Nutrition and Veterinary Public Health, Istituto Superiore di Sanità, 00161 Rome, Italy; elisabetta.suffredini@iss.it

**Keywords:** ddPCR, hepatitis E, food safety, quantification, duplex, internal control

## Abstract

Hepatitis E virus (HEV) represents an emerging risk in industrialized countries where the consumption of contaminated food plays a pivotal role. Quantitative real-time RT-PCR (RT-qPCR) is one of the most suitable methods for the detection and quantification of viruses in food. Nevertheless, quantification using RT-qPCR has limitations. Droplet digital PCR (ddPCR) provides the precise quantification of nucleic acids without the need for a standard curve and a reduction in the effect on virus quantification due to the presence of inhibitors. The objectives of the present work were (i) to develop a method for the absolute quantification of HEV in swine tissues based on ddPCR technology and provide internal process control for recovery assessment and (ii) to evaluate the performance of the method by analyzing a selection of naturally contaminated wild boar muscle samples previously tested using RT-qPCR. The method was optimized using a set of in vitro synthesized HEV RNA and quantified dsDNA. The limit of detection of the developed ddPCR assay was 0.34 genome copies/µL. The analysis of the wild boar samples confirmed the validity of the ddPCR assay. The duplex ddPCR method showed no reduction in efficiency compared to individual assays. The method developed in the present study could represent a sensitive assay for the detection and absolute quantification of HEV RNA in food samples with the advantage of presenting the co-amplification of internal process control.

## 1. Introduction

Foodborne viruses are recognized as a food safety priority by risk assessment experts [1], representing an important issue for the food authorities and the food industry. Different viruses can cause a wide variety of diseases, in particular gastroenteritis or hepatitis. Hepatitis E virus (HEV) is an important cause of acute viral hepatitis in humans worldwide [2], representing an emerging risk in industrialized countries, where transmission through contaminated food plays a key role [3]. In a recent European report, 520 cases of HEV infection were reported in the EU/EEA in January 2024, with an unusual increase of cases in Belgium, Czechia, and Finland when compared to the same time period in 2023 [4].

HEV is classified in the genus *Paslahepevirus*, belonging to the family *Hepeviridae* [5]. It is a hepatotropic, single-stranded positive-sense RNA virus with a genome of 7.2 kb, and eight different genotypes are known (HEV-1 to 8), with five (HEV-1, 2, 3, 4 and 7) infecting humans [5,6]. Genotypes 3 and 4 affect both animals (particularly swine) and humans and are the major cause of hepatitis E in several developed countries [7]. These genotypes are thought to be transmitted through food, mainly via the consumption of undercooked or raw pig, wild boar, and deer meat [8]. Domestic swine and wild boars seem to represent the principal source of zoonotic HEV transmission in Europe and are regarded as the main viral reservoir [8,9]. The virus is mainly excreted through the fecal-oral route, leading to the accumulation and persistence of HEV in the environment. Indeed, environmental contamination, including contamination of organic fertilizers and water, can provide a source for HEV contamination of other types of food, including shellfish, red fruits, and vegetables [10]. Several studies showed the presence of HEV in these food products, confirming the potential risk of HEV transmission [2,11,12,13].

The development of sensitive and reproducible methods for the detection of HEV in food and water samples helps to ensure the safety of these products. The detection of HEV on the basis of its infectivity by cell culture methods is laborious and dependent on a few cell culture-adapted strains [14]. Moreover, cell culture methods are difficult also for the low levels of contamination of food samples.

To date, quantitative real-time RT-PCR (RT-qPCR) is one of the most promising detection methods due to its sensitivity, specificity, and speed in the detection of non-culturable enteric viruses in food. The RT-qPCR assay is considered the gold standard for the quantification of viral RNA. Standard methods for the detection and quantification of hepatitis A virus (HAV) and norovirus (NoV) using RT-qPCR have been developed [15,16]. However, quantification is based on a standard curve constructed from external reference materials. This relative quantification approach has limitations due to differences in the construction of the standard curve, leading to inter-laboratory variations [17]. Several RT-qPCR assays for the detection of HEV have been developed, but they show different levels of sensitivity [18]. Furthermore, the main difficulties in food samples are represented by the low concentrations of the virus and the presence of inhibitory substances present in the sample. It is known that HEV retains infectivity for up to 21 days at 37 °C and after 28 days at room temperature [19]. Generally, stringent thermal treatments eliminate the risk of HEV infection. HEV remains viable after heating to 56 °C for 1 h [20], and cooking to internal temperatures of 71 °C for 20 min is required to fully inactivate the virus [21].

Regarding the impact of food-processing technologies (i.e., fermentation, food aging, curing, drying, smoking, and cooking processes) on viral loads and on HEV virions resistance, few data are available [22]. Although HEV cannot replicate in food, it is relatively stable against various physical-chemical conditions and can remain infectious for long periods on food [14].

Droplet digital PCR (ddPCR) is a new molecular method useful for the precise quantification of nucleic acids without the need for a standard curve [23]. The technology uses a sample dilution analysis and the statistical method of Poisson’s distribution, allowing an absolute quantification of the target. Moreover, being an end-point approach, ddPCR is less subject to variations in terms of amplification efficiency [24] and can reduce the difficulty of quantifying viruses in the presence of inhibitors associated with the type of food matrix [25].

The aim of the present study was to develop a method for the absolute quantification of HEV using the droplet digital PCR technology with internal process control, considered one of the general requirements for viral diagnosis [26]. Moreover, in order to evaluate the sensitivity of the assay, the duplex ddPCR assay was applied to a selection of naturally contaminated wild boar muscle samples from the Apulia and Basilicata regions, derived from hunting activities and intended for human consumption.

## 2. Materials and Methods

### 2.1. ddPCR Assay for HEV Detection

To set up the ddPCR assay for the detection and quantification of HEV, a set of in vitro synthesized HEV RNA (1.0 × 10^4^ genome copies/µL) and a set of a linearized plasmid double-stranded DNA containing the target sequence of the virus (1.40 × 10^5^ g.c./µL) were used. The HEV RNA and dsDNA were quantified spectrophotometrically. The standards were provided by the Istituto Superiore di Sanità (Rome, Italy) as reference materials by transport in dry ice, accompanied by documentation certifying the quantitative values attributed to each material. The ORF3 region of the HEV genome was chosen as the target of the ddPCR, and the primers and probes evaluated were referred to a previous RT-qPCR protocol [27], reported as the most sensitive assay [8], and to protocols derived from it [3,28,29].

Different reaction conditions were evaluated (primer and probe concentrations, reverse transcription temperature, annealing/extension temperature). Two different reverse transcription temperatures (50 °C and 55 °C) and three annealing/extension temperatures (60 °C, 62 °C, and 65 °C) were evaluated. In detail, the optimization of the temperature and concentration of primers and probes were performed using the One-step RT-ddPCR Advanced Kit for Probe (Bio-Rad, Hercules, CA, USA) in a reaction volume of 20 μL (5 μL of Supermix, 2 μL of Reverse transcriptase, 1 μL of DTT (300 mM), 5 μL of the RNA template, and different concentrations of primers (200–900 nM) and probe (100–250 nM)). In Table S1, the different combinations of concentrations tested are listed.

To minimize the errors from pipetting, all reagents were premixed, and then the reaction mixture was emulsified into droplets using 70 μL of droplet generator oil for probes (Bio-Rad, Hercules, CA, USA) with the QX200 droplet generator (Bio-Rad, Hercules, CA, USA).

The generated droplets were carefully transferred to a semi-skirted 96-well PCR plate (Bio-Rad, Hercules, CA, USA). The PCR plate was sealed with pierceable foil and placed onto a C1000 Touch Thermal Cycler (Bio-Rad, Hercules, CA, USA) for PCR amplification. The following thermal cycling conditions were 50 °C or 55 °C for 60 min for reverse transcription, 95 °C for 10 min for enzyme activation, 45 cycles comprising 95 °C for 30 s, 60 °C, 62 °C, or 65 °C for 60 s, 98 °C for 10 min for enzyme deactivation, and a final incubation at 4 °C. After the thermal cycling amplification, the droplets from each well in the plate were read individually with the QX200 Droplet Reader (Bio-Rad, Hercules, CA, USA) using the ddPCR droplet reader oil (Bio-Rad, Hercules, CA, USA). Ten-fold serial dilutions of the in vitro synthesized HEV RNA and dsDNA were evaluated in triplicate.

Data acquisition, analysis, and quantification were carried out with the QuantaSoft™ software version 1.7.4 (Bio-Rad, Hercules, CA, USA). The results of a single well were excluded if the total number of accepted droplets was <10,000. A sample was considered positive if it showed at least three positive droplets, according to previous studies [25,30].

### 2.2. Virus Process Control and Analysis

Mengovirus (strain MC_0_, 1.6 × 10^5^ TCID_50_/mL) provided by the Istituto Superiore di Sanità (Rome, Italy) was used as process control. For the optimization of the ddPCR assay, the RNA was extracted from 10 µL of virus suspension using the NucliSens MiniMAG magnetic system (bioMerieux, Paris, France) according to the manufacturer’s instructions. The eluted RNA (100 µL) was stored at −80 °C until molecular analysis.

The different reverse transcription temperatures and annealing/extension temperatures evaluated for HEV were also tested for the amplification of the mengovirus. The sequence of the primer pairs and probe used in this study were obtained from a previously published study [31], with the final reaction concentrations of 400 nM and 200 nM, respectively. The probe was labeled with the hexachlorofluorescein (HEX)-reported dye at the 5′-end and minor groove binding (MGB) quencher at the 3′-end. A 10-fold serial dilution of the extracted RNA was evaluated in triplicate.

### 2.3. Naturally Contaminated Wild Boar Muscle Samples

In order to evaluate the sensitivity of the assay, the ddPCR protocol was applied to a selection of naturally HEV-contaminated wild boar muscle samples derived from hunting activities and intended for human consumption from the Apulia and Basilicata regions in the year 2022. These samples were previously analyzed using an RT-qPCR method, as previously described [3,32]. In detail, 36 samples were analyzed, of which 18 resulted positive and 18 resulted negative using the RT-qPCR. The portion of 5 g of each sample was spiked with 10 µL of a suspension of mengovirus (strain MC_0_, 1.6 × 10^5^ TCID_50_/mL) prior to the analysis. After viral concentration using TRIzol™ (Invitrogen ThermoFisher Scientific, Frederick, MD, USA) and chloroform, nucleic acids were extracted from 1 mL of the samples’ suspensions using the NucliSens MiniMAG extraction system (bioMerieux, Paris, France) according to the manufacturer’s protocol, and the eluted RNA (100 µL) was stored at −80 °C. The RT-qPCR was carried out using the RNA UltraSense™ One-Step qRT-PCR System kit (Invitrogen, Carlsbad, CA, USA) on a CFX96 Real-Time PCR system thermocycler (Bio-Rad, Hercules, CA, USA). The thermal cycling conditions were 50 °C for 60 min and 95 °C for 5 min, followed by 45 cycles of 95 °C for 15 s, 60 °C for 1 min, and 65 °C for 1 min.

In duplex ddPCR analysis, positive and negative controls were used: HEV RNA in vitro synthesized (dilution 1 × 10^3^ g.c./µL) as external amplification control and nuclease-free water as negative control.

## 3. Results

### 3.1. Duplex ddPCR Assay for Simultaneous Detection of HEV and Mengovirus

#### 3.1.1. HEV Assay Optimization

The assay optimization tests allowed us to define the best conditions (temperature/reagent concentration) and the limit of detection (LOD) of the method in ddPCR. In detail, the reverse transcription temperature of 50 °C showed the most suitable one, resulting in an increased sensitivity of the test, regardless of the other conditions evaluated. This result was found for all the different primer/probe concentration conditions evaluated.

**Figure 1:**
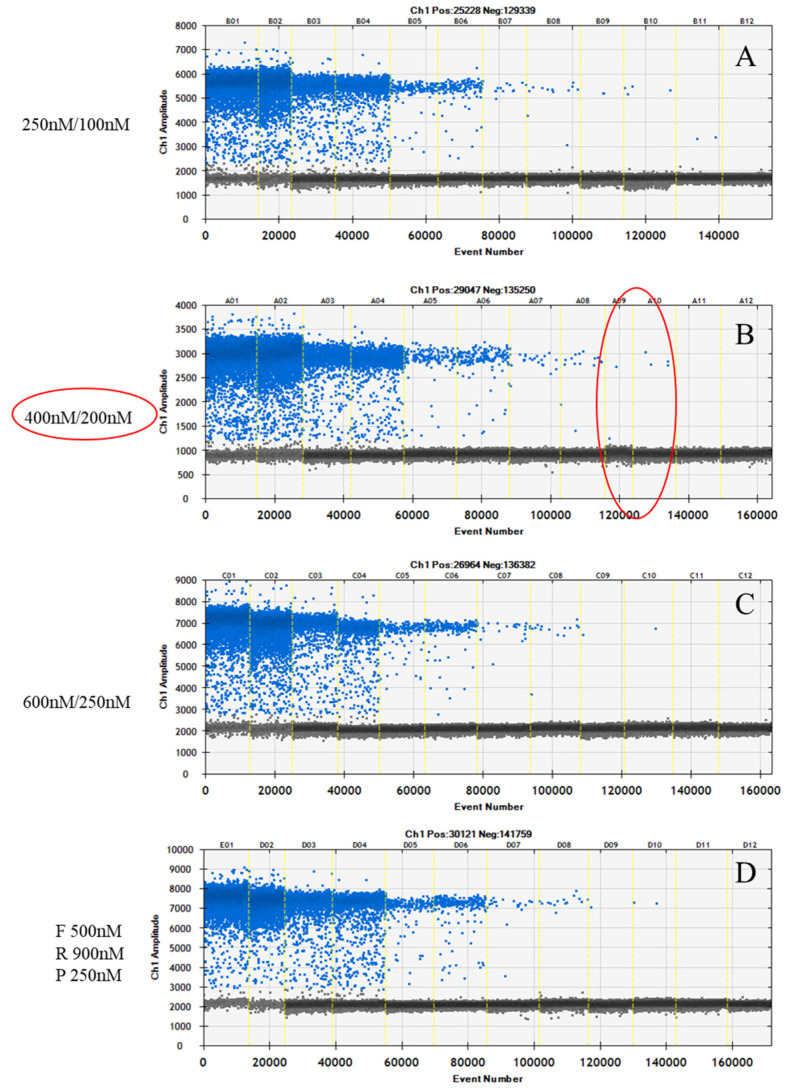
Optimization of the concentration of the primers and probe of HEV ddPCR. Results for the analysis of the 10-fold serial dilutions of the in vitro synthesized HEV RNA: (**A**) primers 250 nM, probe 100 nM; (**B**) primers 400 nM, probe 200 nM; (**C**) primers 600 nM, probe 250 nM; (**D**) primer forward (F) 500 nM, primers reverse (R) 900 nM, probe (P) 250 nM. The combination of 400–200 nM, respectively, for primers and probe enabled the detection of the dilution of in vitro synthesized HEV RNA equal to a theoretical concentration of 1 g.c./µL (red circle). Positive droplets (blue) and negative droplets (grey) are shown.

Regarding the different primers/probe concentration conditions, the combinations 250–100 nM and 400–200 nM, respectively, for primers and probe allowed for the detection of the dilution equal to a theoretical concentration of 1 g.c./µL (Figure 1). The condition 400 nM for primers forward and reverse and 200 nM for the probe was chosen for further analysis, providing more discriminating results with correct separation of negative and positive droplets.

**Figure 2:**
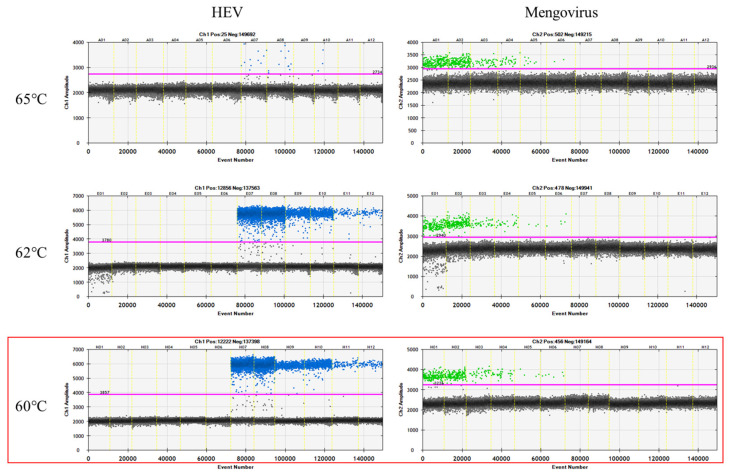
Results of analysis for the optimization of the annealing/extension temperature for the detection of HEV and process control (mengovirus). The temperature of 60 °C (red box) showed the most suitable, resulting in a better separation of positive (blue for HEV and green for mengovirus) and negative (gray) droplets for both targets.

In order to determine the optimum annealing/extension temperature, three temperatures (60 °C, 62 °C, and 65 °C) were evaluated. At the temperature of 60 °C, the negative and positive droplets were separated distinctly. The same conditions provided similar results for the analysis of mengovirus (Figure 2).

In the evaluation of the HEV/mengovirus duplex assay, no reduction in efficiency due to a competition of the two reactions was detected.

In Table 1, the reaction mixture of duplex ddPCR assay is reported.

Moreover, in order to evaluate the interference between the two viruses, the duplex assay was checked also using RT-qPCR, analyzing the in vitro synthesized HEV RNA, the extracted RNA of mengovirus, and two tissue samples, one positive and one negative for HEV. The results confirmed that the data obtained by ddPCR showed no interference. In Figure S1, the results of the analysis are shown.

#### 3.1.2. Performance of the HEV ddPCR Assay

Following the optimization of the assay for the detection of HEV by ddPCR, the performance of the method was evaluated in relation to the RT-qPCR assay developed by Di Pasquale et al. [3]. The analysis was conducted by preparing 10-fold serial dilutions of the in vitro synthesized HEV RNA and dsDNA in triplicate until the theoretical dilution equal to 1 g.c./µL was reached. While using the RT-qPCR, the nucleic acids were detected at the concentration of 10 g.c./µL; using ddPCR, it was possible to detect at the lowest dilution (1 g.c./µL). In Figure S2, the amplification plot obtained on HEV RNA positive control by RT-qPCR is shown. Moreover, the quantification using ddPCR was lower compared with the spectrophotometric determination up to 1 log, with a linear response for all dilutions remaining in the same order of measurement in terms of logarithmic reduction. In fact, the concentration detected and calculated with the QuantaSoft™ software version 1.7.4 for the last dilution is equal to 0.34 g.c./µL, thus resulting in the LOD of the developed assay.

In all series, the first dilutions, the most concentrated ones, were not determined due to system saturation (high concentration of nucleic acids), while the lower concentration dilutions provided more discriminating results with a correct separation of negative and positive droplets.

### 3.2. Analysis of Naturally Contaminated Wild Boar Muscle Samples

The ddPCR analysis of the wild boar samples previously analyzed using RT-qPCR confirmed the positivity and negativity of the samples. The differences found were related to quantification. As for the analysis of the HEV standard, a difference of 1 log_10_ was detected between the RT-qPCR and ddPCR results, as well as a different quantification depending on the viral titer of the sample. In fact, ddPCR quantified with greater precision the samples with viral titers below 10 g.c./µL. On the contrary, for a sample with a high viral load (>10^5^ g.c./µL), the ddPCR was not able to quantify due to a saturation of the system. Table 2 shows the results for the 18 positive samples analyzed. Regarding the process control virus, this was detected in all analyzed samples (range number of positive droplets for mengovirus: min 3—max 1064, mean 199), except for a sample (ID 25874/590) with a high HEV viral load, where all generated droplets included one or more copies of HEV RNA (Figure S3). In Figure S4, a comparison between the RT-qPCR curves and the ddPCR rain plots for some significant wild boar muscle analyzed samples is shown.

## 4. Discussion

Food transmission of the hepatitis E virus is mainly due to the consumption of raw or undercooked meat from infected animals such as pigs or wild boars [8]. In the absence of universal cell culture systems for HEV, in addition to the unsuitability of these techniques for control activities due to analytical times, molecular diagnostic methods represent a valid tool capable of providing useful information on the presence and prevalence of HEV in food. Currently, standardized methods for the detection of HEV in food are not yet available. The standardization process began in the ISO/TC 34/SC 9 WG 31 working group and will lead to a procedure in the following years. To date, various molecular methods are available in the literature for HEV detection and quantification, but they differ from each other in terms of sensitivity and specificity. Among these, the method developed by Jothikumar et al. [27] is reported to be the most sensitive RT-qPCR assay for HEV detection [8]. The quantification of viral DNA is performed by reference to a standard curve generated from a dilution series of linearized dsDNA standards (external reference materials) carrying the relevant target sequences. This quantification approach has limitations due to differences in the construction of the standard curve (i.e., certified dsDNA standards are not available), leading to inter-laboratory variations [17]. Furthermore, the main difficulties in the detection of viruses in food are represented by the low viral load and the possible presence of inhibitory substances, with consequences on the amplification efficiency. Therefore, it is important to use process control to verify the different phases of the analytical process. To date, few duplex RT-qPCR assays for the quantitative detection of HEV, including an internal control, are available [29,33,34].

Droplet digital PCR enables the absolute quantification of a single nucleic acid molecule and provides advantages compared with other molecular methods in terms of precision, accuracy, and sensitivity for samples with a low viral load [35,36]. In the last few years, droplet digital PCR has been demonstrated to give precise quantification of nucleic acids within wide food safety applications, including meat and milk authenticity [37,38], and detection of intracellular pathogens difficult to detect [39].

Even though previous studies applied the ddPCR for the quantification of HEV, none provided a co-amplification of process control [17,40,41,42,43].

In the present work, a duplex ddPCR assay for detecting HEV that presents an internal control to verify any losses of the virus during the analytical process or inhibition was developed.

The results of the present study confirmed the validity of the assay developed by Jothikumar et al. [27], with the subsequent probe modification reported by Garson et al. [28] representing the most sensitive method. This method, optimized in ddPCR here, showed a LOD of 0.34 g.c./µL and showed the best separation of positive and negative droplets. Moreover, the nucleic acid concentrations of reference materials (in vitro synthesized HEV RNA and dsDNA) quantified by ddPCR did not match with spectrophotometric measurements. This result is in agreement with previous studies [17,44,45]. This difference is due to errors in the spectrophotometric measurement of nucleic acids, which lead to an overestimation of the number of genomic copies of the standards [44,46,47].

Many studies have highlighted the problem of using standards for the quantification of nucleic acids by RT-qPCR, as they can themselves be a source of error. Therefore, droplet digital PCR represents a promising technology, with the advantage of quantifying nucleic acids without the need for a standard curve. It could also be useful in reducing variations in quantitative data obtained from different laboratories. Recently, an international inter-laboratory study comparing digital PCR to ISO-standardized RT-qPCR assays for the detection of norovirus GI and GII in oyster tissue has proven that the digital PCR assay is likely to increase uniformity of quantitative results between laboratories [48].

At the same time, ddPCR represents a valid diagnostic tool for the evaluation of samples with low concentrations of HEV RNA.

The analysis of the wild boar samples confirmed the validity of the ddPCR assay.

In fact, all samples naturally contaminated with HEV tested positive using RT-qPCR; HEV RNA was also detected using ddPCR. The two methods differ in the quantities of genomic copies calculated. In fact, even for these samples, as for the standards, ddPCR determined a genomic copy number lower than that determined by RT-qPCR, especially for high viral load samples. Furthermore, the data confirmed the improved quantification of ddPCR for samples with a low HEV RNA concentration. On the contrary, ddPCR has the limitation of not being able to quantify the nucleic acids for high viral load samples, except through dilution of the sample itself.

Although molecular assays do not provide information on the infectivity of the virus, the presence of HEV nucleic acid in food portions, even at low concentrations, cannot be overlooked. The infectious dose of HEV for humans is not well defined, and it is possible that the low viral load detected may or may not contain infectious particles [42]. The development of efficient and reproducible cell culture methods for HEV is needed to facilitate the acquisition of quantitative data on the infectivity, inactivation, and survival of HEV in food and in the environment.

In conclusion, the ddPCR method developed in the present study represents a sensitive and accurate assay for the absolute quantification of HEV RNA in food samples, with the advantage of presenting the co-amplification of an internal control capable of monitoring the whole analytical process. Moreover, it has been estimated that a duplex ddPCR assay was less expensive than two separate RT-qPCR assays for one sample (about 6 Euros for ddPCR compared with about 8 Euros for RT-qPCR), and mainly the dsDNA dilution series for the quantification curve adds to the total costs [48]. The developed method can provide a valid diagnostic tool for the accurate detection of the HEV viral load for the prevention and control of the presence of this virus in food. The present study presents some limitations, which represent, at the same time, aims for further studies: (i) to test the assay analyzing artificially contaminated samples of several food types, (ii) to apply the method to a larger number of samples, and (iii) to validate the assay with an inter-laboratory study.

## Figures and Tables

**Table 1:** Reagents and composition of the duplex ddPCR assay for simultaneous detection of HEV and mengovirus.

Reagents	Final Concentration	Volume (µL)
Supermix	1×	5
HEV FW (20 μM)	400 nM	0.4
HEV REV (20 μM)	400 nM	0.4
HEV Probe (10 μM) ^1^	200 nM	0.4
Mengo FW (20 μM)	400 nM	0.4
Mengo REV (20 μM)	400 nM	0.4
Mengo Probe (10 μM) ^2^	200 nM	0.4
DTT 300 mM	15 nM	1
RT mix	(20 U/μL)	2
Nuclease-Free Water	-	4.6
RNA Template	-	5

^1^ Probe labeled 5′ FAM-MGB 3′. ^2^ Probe labeled 5′ HEX-MGB 3′.

**Table 2:** Results of quantification using RT-qPCR and ddPCR of naturally contaminated wild boar samples.

Sample ID	RT-qPCR		ddPCR	
	(avg g.c./µL) *	SD	(avg g.c./µL) **	SD
25015	5.02 × 10^3^	25.19	6.03 × 10^2^	2.08
25864/169	2.67	0.09	0.08	0.06
25964/167	7.67	0.31	0.62	0.12
25874/590	2.61 × 10^5^	935.67	1.00 × 10^6^	0.00
26430/23	9.15 × 10	3.32	1.43 × 10^2^	2.01
26628	7.61 × 10^2^	14.48	9.0 × 10	0.51
26659/53	9.38 × 10	1.09	3.41 × 10	0.48
26889/28	2.06	0.02	0.35	0.09
26889/30	2.81 × 10^2^	29.35	6.33 × 10	0.50
26889/31	9.19 × 10^2^	28.82	1.30 × 10^2^	2.01
26889/27	8.34 × 10	10.76	2.3	0.17
26901	0.44	0.29	0.14	0.07
25325/904	7.67	0.12	0.7	0.11
25438/4	0.13	0.26	0.17	0.08
24553	1.48	0.56	3.9	0.23
25968/903	0.05	0.05	0.2	0.08
25869/901	6.44 × 10^3^	2074.72	6.19 × 10^3^	43.18
3888	7.73	0.15	1.6	0.15

* The quantification was carried out by analyzing the sample in quadruplicate. ** The quantification was carried out by analyzing the sample in triplicate. SD, standard deviation.

## Data Availability

The original contributions presented in the study are included in the article; further inquiries can be directed to the corresponding author.

## Supplementary Materials

The following supporting information can be downloaded at: https://www.mdpi.com/article/10.3390/v16030413/s1, Table S1: Detail of the different concentrations of primers and probe tested for ddPCR assay optimization, Figure S1: Results of analysis for the duplex assay HEV/process control (mengovirus) using RT-qPCR, Figure S2: Standard curve obtained on HEV RNA positive control (in vitro synthesized) using RT-qPCR, Figure S3: ddPCR plot for the wild boar muscle samples analyzed, Figure S4: Comparison between the RT-qPCR curves and the ddPCR rain plots for the most significant wild boar muscle analyzed samples.
