# Supplementary material for: Duplex Droplet Digital PCR Assay for Quantification of Hepatitis E Virus in Food
Source: Viruses. 2024 Mar 7;16(3):413. doi: 10.3390/v16030413 (PMC10975721; doi:10.3390/v16030413)
Supplement: Supplementary file 1 [file viruses-16-00413-s001.zip › viruses-2878513-supplementary.pdf]

Table S1. Detail of the different concentrations of primers and probes tested for ddPCR assay optimization.

Concentration	Reference
200 nM primers<br>100 nM probe [1]	Jothikumar et al., 2006 [27]
400 nM primers<br>200 nM probe [2]	Garson et al., 2012 [28]
600 nM primers<br>250 nM probe [3]	Martin-Latil et al., 2012 [29]
500 nM primer forward<br>900 nM primer reverse<br>250 nM probe [2]	Di Pasquale et al., 2019 [3]

[1] Probe labeled 5′-6-carboxy fluorescein fluorophore (FAM) and 3′-black hole quencher (BHQ). [2] Probe labeled 5′-6-carboxy fluorescein fluorophore (FAM) and 3′-minor groove binding (MGB) quencher. [3] Probe labeled 5′-carboxy-X-rhodamine fluorophore (ROX) and 3′-black hole quencher (BHQ).

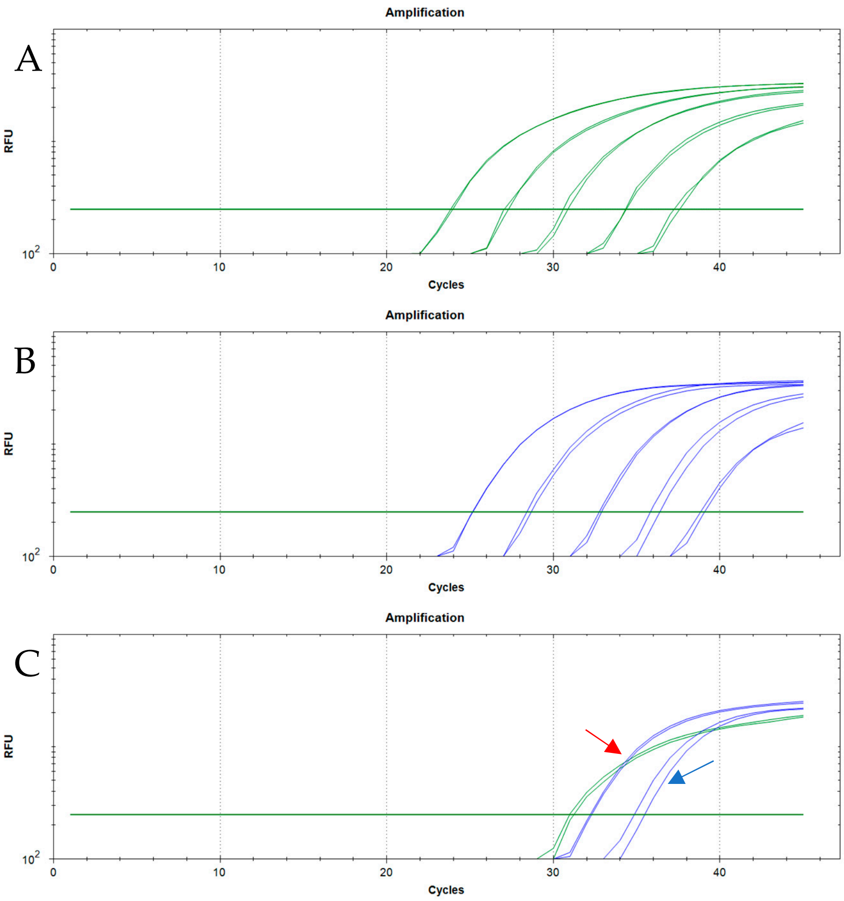

Figure S1. Results of analysis for the duplex assay HEV/process control (mengovirus) by RT-qPCR. (A): analysis of a 10-fold serial dilution of the in vitro synthetized HEV RNA. In green the fluorescence for the target HEV and no fluorescence for mengovirus; (B): analysis of a 10-fold serial dilution of the extracted RNA of mengovirus. In blue the fluorescence for the target mengovirus and no fluorescence for HEV; (C): analysis of two samples, one positive sample (red arrow) and one negative (blue arrow) for HEV. The red arrow indicates the fluorescence signals for both HEV and process control, while the blue arrow indicates the fluorescence signal only for the process control. The analysis was carried out in duplicate.

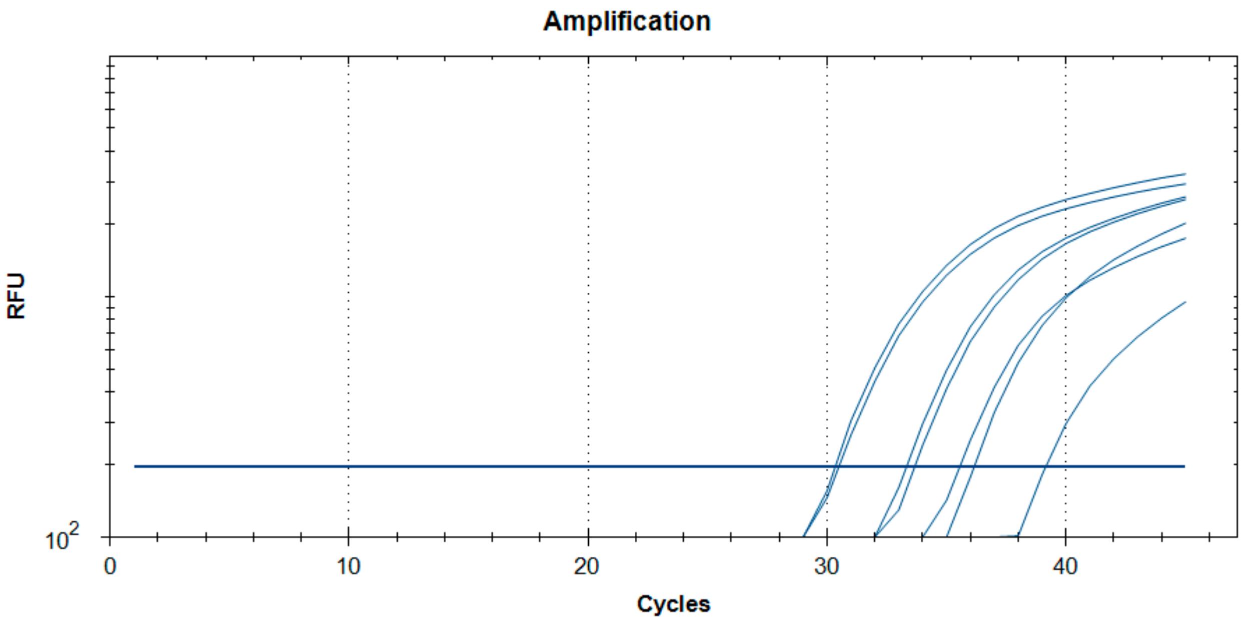

Figure S2. Standard curve obtained on HEV RNA positive control (in vitro synthesized) by RT-qPCR. A 10-fold serial dilution of the positive control (1.0×10^4 genome copies/μl) was analyzed according the protocol previously described [3]. The nucleic acids were detected up to the concentration of 10 g.c./μl.

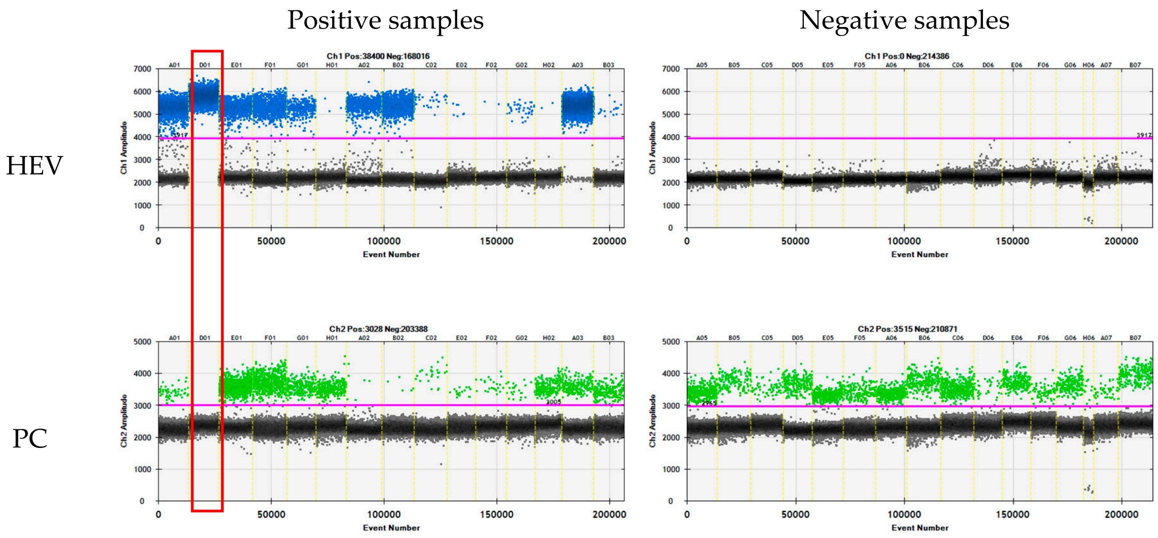

Figure S3. ddPCR plot for the wild boar muscle samples analyzed. The left panels show the results for the positive samples and the right panels show the results for the negative samples. The target HEV (blue droplets) was detected only in the positive samples, whilst the positive control (mengovirus, green dropltes) was detected in all the samples except for samples with a high HEV viral load (sample ID 25875/590), where all generated droplets included one or more copies of HEV RNA (red box).

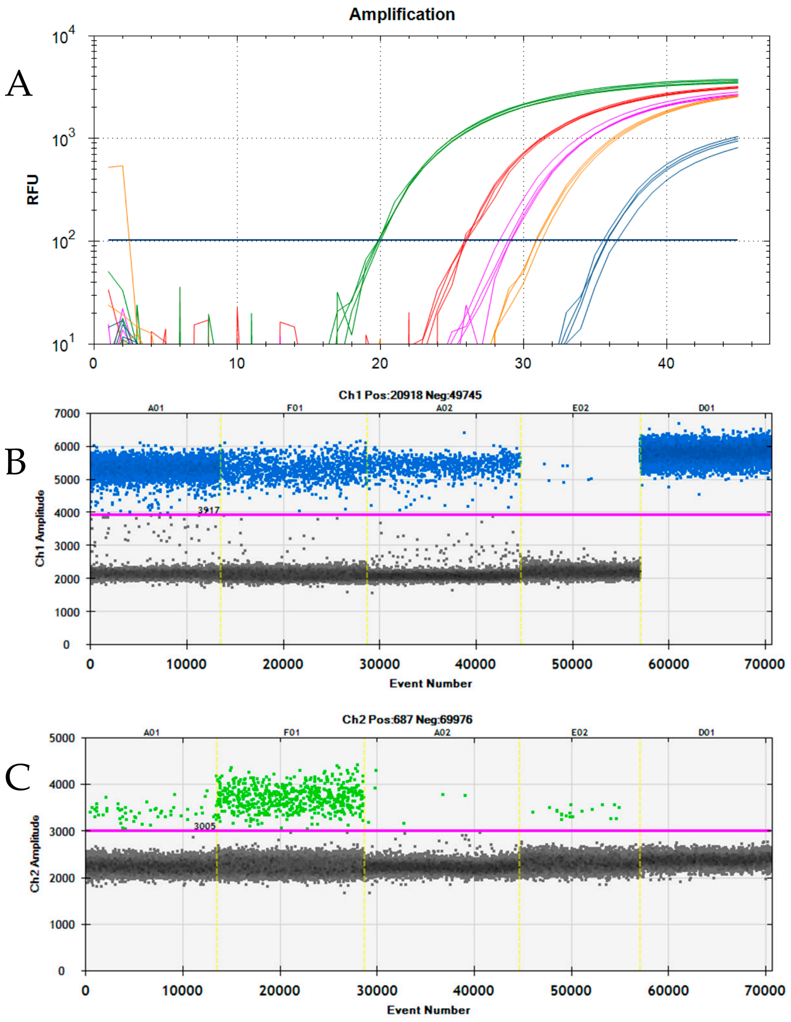

Figure S4. Comparison between the RT-qPCR curves and the ddPCR rain plots for the most significant wild boar muscle analyzed samples. (A): RT-qPCR curves for samples ID 25015 (red), ID 26628 (pink), ID 26889/30 (orange), ID 25325/904 (blue), and ID 25875/590 (green); (B) and (C): correspondent ddPCR rain plots for samples ID 25015 (well A01), ID 26628 (well F01), ID 26889/30 (well A02), ID 25325/904 (well E02), and ID 25875/590 (well D01). In the samples with a high HEV viral load (sample ID 25875/590), no positive droplets for the process control were detected (well D01) because all generated droplets included one or more copies of HEV RNA.
